# Undiagnosed Schizencephaly Presenting as Breakthrough Seizures

**DOI:** 10.5811/cpcem.20922

**Published:** 2024-08-16

**Authors:** John Coacci, Peter Viccellio

**Affiliations:** Stony Brook Medicine, Department of Emergency Medicine, Stony Brook, New York

**Keywords:** schizencephaly, epilepsy, seizure, neurology, neurosurgery

## Abstract

**Case Presentation:**

A 19-year-old male presented for evaluation of breakthrough seizures after inability to refill his medication following recent immigration from Haiti. Previously, the patient had never received neuroimaging due to financial constraints and resource scarcity. Computed tomography and magnetic resonance imaging obtained in the emergency department was significant for large right frontoparietal open-lip schizencephaly with mass effect, a rare congenital neurologic disorder previously undiagnosed in this patient with intractable epilepsy.

**Discussion:**

Schizencephaly is a rare congenital neurodevelopmental disorder, which has diverse presentations ranging from intractable epilepsy to variable degrees of neurocognitive dysfunction. Treatment is generally focused on seizure management and rehabilitation. Furthermore, emergency physicians must be cognizant of patients with social determinants of health, which may have formerly prevented thorough evaluation and aid in appropriate treatment of these patients.

## CASE PRESENTATION

A 19-year-old male presented for evaluation of breakthrough seizures after inability to refill his medication following recent immigration from Haiti. He had previously been diagnosed with an unspecified seizure disorder and prescribed diazepam daily. Neuroimaging was never obtained due to financial constraints and resource scarcity. Computed tomography revealed large right frontoparietal open-lip schizencephaly with right-to-left midline shift (Image A). Additional anomalies included absence of the septum pellucidum and communication of the lateral ventricles. Subsequent magnetic resonance imaging elucidated areas of gray-white matter heterotopia and polymicrogyria, and partial fusion of the fornix concerning for lobar holoprosencephaly (Image B).

The patient was admitted to the neurology service, where video electroencephalogram revealed bihemispheric dysfunction with epileptogenic potential from the left temporal region. The patient was started on an appropriate anti-epileptic regimen and given neurosurgery referral to discuss elective ventriculoperitoneal shunt placement.

## DISCUSSION

Schizencephaly is a rare congenital disorder characterized by the presence of a cleft in the cerebral hemisphere lined with heterotrophic gray matter, extending from the surface of the pia mater to the lateral ventricles. “Closed-lip” (type I) schizencephaly contains clefts that do not communicate with the ventricular system, while “open-lip” (type II) schizencephaly contains clefts that communicate with the ventricular system. The incidence is estimated at 1.54/100,000 live births.^1^–^4^ Patients may present with intractable epilepsy and varying degrees of neurocognitive dysfunction.^1^,^3^ Associated congenital anomalies may include agenesis of the corpus collosum or septum pellucidum.^1^,^5^ Treatment is targeted toward rehabilitation and seizure management. Surgery, including shunt placement, is indicated in cases of increased intracranial pressure secondary to hydrocephalus.^4^,^5^

For patients with schizencephaly, early diagnosis and treatment can aid in attaining better neurodevelopmental outcomes.^4^ Physicians must be cognizant of patients with social determinants of health, which may have impeded the ability to obtain thorough diagnostic evaluation, and aid in obtaining appropriate treatment resources.

**CPC-EM Capsule**

What do we already know about this clinical entity?

*Schizencephaly is a rare neurodevelopmental disorder often presenting as intractable seizures and varying degrees of neurocognitive delay.*

What is the major impact of the image(s)?

*In this patient with an established seizure disorder who previously could not obtain neuroimaging, computed tomography revealed large, right open-lip schizencephaly.*

How might this improve emergency medicine practice?

*Emergency physicians must be cognizant of social determinants of health when evaluating patients, as some may have experienced significant barriers to proper care.*

## Figures and Tables

**Image:**
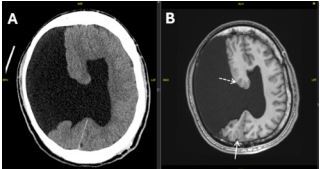
(A) Large right frontoparietal open-lip schizencephaly demonstrated on non-contrast computed tomography. (B) T1-weighted non-contrast magnetic resonance imaging demonstrates heterotopic gray matter (dashed arrow) and polymicrogyria (solid arrow).
